# Double Perovskite La_2_MnNiO_6_ as a High‐Performance Anode for Lithium‐Ion Batteries

**DOI:** 10.1002/advs.202300506

**Published:** 2023-04-21

**Authors:** Chang Zhang, Yue Zhang, Zhiwei Nie, Cong Wu, Tianyi Gao, Nan Yang, Yi Yu, Yuanyuan Cui, Yanfeng Gao, Wei Liu

**Affiliations:** ^1^ School of Physical Science and Technology ShanghaiTech University Shanghai 201210 P. R. China; ^2^ Shanghai Key Laboratory of High‐resolution Electron Microscopy ShanghaiTech University Shanghai 201210 P. R. China; ^3^ School of Materials Science and Engineering Shanghai University Shanghai 200444 P. R. China; ^4^ Key Laboratory of Advanced Energy Materials Chemistry (Ministry of Education) Nankai University Tianjin 300071 P. R. China

**Keywords:** lithium‐ion batteries, anode material, double perovskite, rate capability, cycling stability

## Abstract

Traditional lithium‐ion batteries cannot meet the ever‐increasing energy demands due to the unsatisfied graphite anode with sluggish electrochemical kinetics. Recently, the perovskite material family as anode attracts growing attention due to their advantages on specific capacity, rate capability, lifetime, and safety. Herein, a double perovskite La_2_MnNiO_6_ synthesized by solid‐state reaction method as a high‐performance anode material for LIBs is reported. La_2_MnNiO_6_ with an average operating potential of <0.8 V versus Li^+^/Li exhibits a good rate capability. Besides, the Li|La_2_MnNiO_6_ cells perform long cycle life without decay after 1000 cycles at 1C and a high cycling retention of 93% is observed after 3000 cycles at 6C. It reveals that this material maintains stable perovskite structure with cycling. Theoretical calculations further demonstrate the high electronic conductivity, low diffusion energy barrier, and structural stability of the lithiated La_2_MnNiO_6_. This study highlights the double perovskite type material as a promising anode for next‐generation batteries.

## Graphical abstract

Double perovskite type oxide La_2_MnNiO_6_, for the first time, is used as battery anode material, and a reversible Li^+^ insertion/extraction process is proved by in situ XRD and HR‐TEM. The LMNO anode can deliver long cycle life and good rate capability. Additionally, theoretical calculations further demonstrate the high electronic conductivity, low diffusion energy barrier, and structural stability of LMNO.

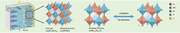

## 1 Introduction

Lithium‐ion batteries (LIBs), as most used energy storage device, significantly facilitate peoples’ life since the first report in 1990s. However, traditional LIBs could not meet the ever‐increasing energy and safety demands for electric vehicles (EVs) up to now.^[^ ^1^, ^2^, ^3^, ^4^, ^5^ ^]^ The low insertion/extraction kinetics of the graphite anode restricts the Li‐ion diffusion, resulting in a weak rate capability and fast‐charging performance. The low working potential (≈0.1 V versus Li^+^/Li) of graphite is close to lithium plating potential, possibly causing “dead” Li and lithium dendrite growth, which may cause fast capacity decay and serious safety issues.^[^ ^6^, ^7^, ^8^ ^]^ Spinel Li_4_Ti_5_O_12_ (LTO) is another widely used anode materials because of good rate capability, whereas low specific capacity (<170 mA h g^−1^) and high operation potential (1.55 V versus Li^+^/Li) bring corresponding low energy density, which greatly restrict in its practical applications.^[^ ^9^, ^10^, ^11^ ^]^ Therefore, it is significant to explore superior anode materials with dual functions of high capacity and safe lithiation potential for further boosting the performance of LIBs.

Various new materials with superior performance have been reported in recent years, concluding Si, Si‐C, P, MXenes, Zeolites, MOFs and metal oxides/sulfides.^[^ ^12^, ^13^, ^14^, ^15^, ^16^, ^17^, ^18^, ^19^, ^20^, ^21^, ^22^, ^23^, ^24^, ^25^, ^26^, ^27^, ^28^, ^29^, ^30^, ^31^ ^]^ For example, Liu et al.^[^ ^21^ ^]^ found rock salt Li_3_V_2_O_5_ can achieve over 1000 cycles with negligible capacity decay and exhibits exceptional rate capability. Grey group^[^ ^23^ ^]^ reported micrometer‐scaling niobium tungsten oxides particles, which achieved ultrahigh rate performance by designing appropriate 3D crystal structures and stable host structures. Niobium tungsten oxides exhibit the average operation potentials are as high as 1.6–1.9 V, which possibly could not meet the requirement of high energy density. In addition to these materials, perovskite oxide family attracts growing attention due to their advantages on rate capability, lifetime, and safety. In addition to these materials, perovskite oxide family attracts growing attention due to their advantages on rate capability, lifetime, and safety.^[^ ^9^, ^32^, ^33^ ^]^ Perovskite ABO_3_ structure could be considered as from the parent ReO_3_ structure type, with the large A cations at the framework center to stabilize the structure.^[^ ^23^ ^]^ Very recently, Liu et al.^[^ ^32^ ^]^ reported the synthesis of perovskite SrVO_3_ as anode material and revealed Li^+^ storage mechanism of intercalation type reaction. The SrVO_3_ could offer a high specific capacity as well as high rate‐capability at average operation potential of ≈0.9 V versus Li^+^/Li. Similarly, Hirano group^[^ ^33^ ^]^ synthesized bulk LiYTiO_4_ with layered perovskite structure, displaying low work potential and ultrahigh‐rate performance. Moreover, halide perovskite family is another important part of perovskite anode materials, such as metal halide perovskite CsPbX_3_^[^ ^34^, ^35^ ^]^ and organic–inorganic halide perovskites CH_3_NH_3_PbX_3_ (X = Cl, Br, and I).^[^ ^36^ ^]^ However, SrVO_3_ was prepared by using facile solution combustion and followed by a thermal reduction process in H_2_ atmosphere.^[^ ^32^ ^]^ And LiYTiO_4_ came from NaYTiO_4_ by ion‐exchange in molten LiNO_3_ for several times.^[^ ^33^ ^]^ These processes are complex with increased cost and restrict the large‐scale applications.

In this work, we report a double perovskite La_2_MnNiO_6_ (LMNO) as anode material with high performance and safety working potential, which was synthesized by a simple and rapid process of ball‐milling mixture followed by calcination. The LMNO anode can deliver superior electrochemical performances, including long cycle life and good rate capability. The Li^+^ intercalation/deintercalation revolution mechanism in LMNO was systematically investigated. The results show the lithiation‐LMNO and delithiation‐LMNO maintain stable perovskite structure with cycling. Additionally, theoretical calculations further demonstrate the high electronic conductivity, low diffusion energy barrier, and structural stability of the double perovskite LMNO.

## 2 Result and Discussion

**Figure 1:**
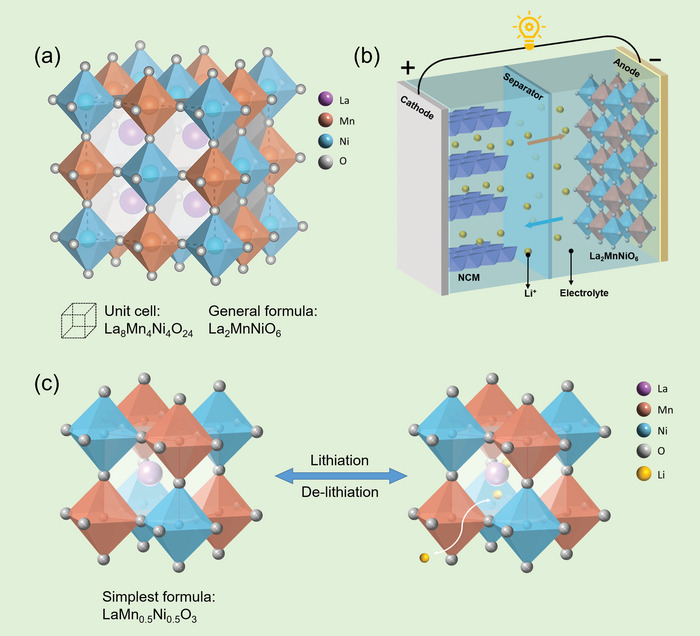
a) Structure schematic illustration of the double perovskite La_8_Mn_4_Ni_4_O_24_ (La_2_MnNiO_6_, LMNO). b) Schematic illustration of LMNO|NCM Li‐ion battery. c) Schematic illustration of lithiation/de‐lithiation process in LaMn_0.5_Ni_0.5_O_3_ cell.

Perovskite type anodes were considered as a kind of promising electrode materials and have been reported several times recently.^[^ ^9^, ^32^, ^33^ ^]^ However, double perovskite materials have not been studied systematically as anode for LIBs up to now. The schematic illustration of double perovskite LMNO crystal structure is given in **Figure** **1**a. The model drawn by black dashed line represents the atomic configuration of La_8_Mn_4_Ni_4_O_24_, which is always written as La_2_MnNiO_6_ (named as LMNO in this paper). Figure 1b represents the illustration of full cells based on LMNO anode. If described in a standard perovskite ABO_3_ cell (Figure 1c), the larger La cation occupies A‐site, B‐site is occupied alternately by smaller Mn ion and Ni ion.^[^ ^37^, ^38^, ^39^ ^]^ During the lithiation process, Li^+^ ions insert the perovskite structure interstice to form a solid solution, and the reversible desertion behavior occurs in delithiation process.

**Figure 2:**
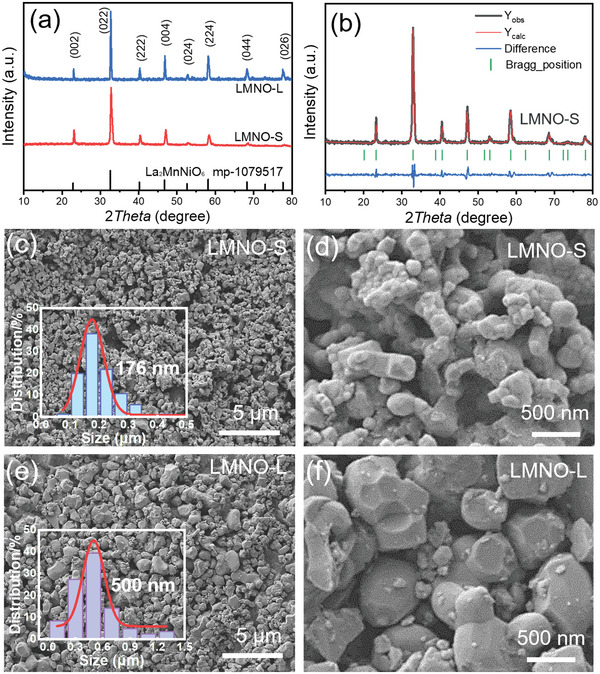
Phase and morphology characterization of LMNO with various particle sizes. a) XRD patterns of LMNO‐S and LMNO‐L. b) Pawley analysis results of XRD patterns for La_2_NiMnO_6_. Space group: Fm3_m; crystal structure: cubic with *a* = 0.777 nm; *R*_p_ = 9.2%, *R*_wp_ = 11.9%. c,d) SEM images of LMNO‐S, the inset in (c) is diameter distribution of LMNO‐S. e,f) SEM images of LMNO‐L, the inset in (e) is diameter distribution of LMNO‐L. The red curves in (c,e) are the fitting line by Gauss Function.

LMNO is synthesized by a simple solid‐state reaction method.^[^ ^37^ ^]^ After ball‐milling and drying, the mixture is calcinated at various temperatures (1200 and 1400 °C) to obtain LMNO particles with different grain diameters. The crystal structure of LMNO was determined by X‐ray diffraction method (XRD). The XRD spectra (**Figure** **2**a) demonstrate the typical sharp diffraction peaks at 2*θ* = 22.8° (002), 32.6° (022) and 40.2° (222), which match well with double perovskite type La_2_MnNiO_6_ (material project, mp‐1079517, cubic). Particle morphology and grain diameter distribution were obtained from scanning electron microscopy (SEM) images, and Gauss function was employed to fit the histograms plot (Figure 2c–f). LMNO calcinated at 1200 °C has a smaller average size of 176 nm, named LMNO‐S. LMNO calcinated at 1400 °C shows a larger average size of 500 nm, named LMNO‐L. Due to the higher calcination temperature, LMNO‐L exhibits sharper diffraction peak and smaller half peak width. Moreover, Pawley refinement of XRD pattern of LMNO‐S was conducted to further confirm the phase structure. Experimental, calculated and difference XRD patterns after Pawley refinement of LMNO‐S were given in Figure 2b, indicating the phase of double perovskite structure (*R*_p_ = 9.2%). X‐ray photoelectron spectroscopy (XPS) technique is employed to verify chemical composition and element valence state of LMNO. The XPS spectra of LMNO (Figure S1a–c, Supporting Information) reveal the classical perovskite feature by comparing with previous reports.^[^ ^40^, ^41^, ^42^, ^43^, ^44^ ^]^

**Figure 3:**
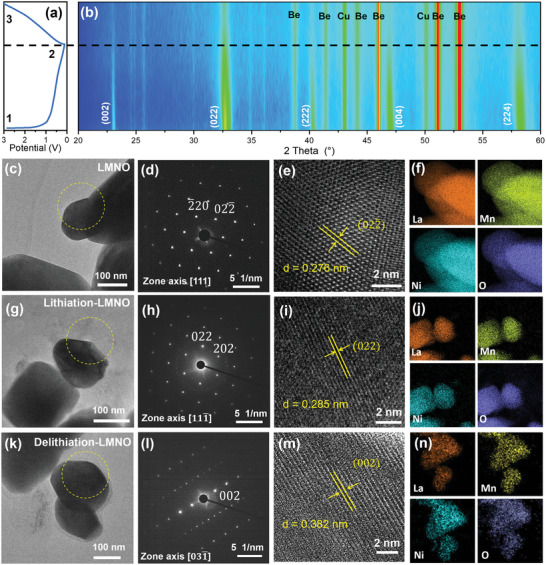
Phase structure evolution of LMNO. a,b) Contour mapping of in situ XRD during the first discharge–charge process. c–e) TEM image, SAED pattern (viewed along zone axis [111]) and HR‐TEM image of LMNO. f) Corresponding EDS elemental mapping images of La, Mn, Ni, and O elements. g–i) TEM image, SAED pattern (viewed along zone axis [$11\bar{1}$]), and HR‐TEM image of lithiation‐LMNO. j) EDS elemental mapping images. k–m) TEM image, SAED pattern (viewed along zone axis [$03\bar{1}$]), and HR‐TEM image of delithiation‐LMNO. n) EDS elemental mapping images.

To further reveal Li^+^ insertion/extraction mechanism, in situ XRD and ex situ XRD are employed to observe the change of LMNO‐S crystal structure. The in situ XRD cell was assembled as described in schematic diagram (Figure S2a, Supporting Information), and the in situ XRD patterns were collected during the first discharge–charge process. An irreversible process occurred in the first discharging–charging curve of **Figure** **3**a, which could be attributed to the formation of amorphous solid electrolyte interphase (SEI) layer on the surface of LMNO particles. As shown in Figure 3b, except for the peaks of Cu mesh current collector and Be window, the intensity of peaks of LMNO‐S electrode reduced slowly during the lithiation process, which also correspond to the SEI growth process. And there is no appearance of new peaks or disappearance of original peaks at a later delithiation process when comparing with LMNO‐S (Figure 2a, and Figure S2b, Supporting Information), indicating that the perovskite structure evolves by a simple solid‐solution mechanism.^[^ ^32^, ^33^ ^]^ This phenomenon proves that the reversible Li^+^ insertion/extraction process occurs in double perovskite type LMNO without any structural destruction.

Moreover, high‐resolution transmission electron microscopy (HR‐TEM) and corresponding energy dispersive spectrometry (EDS) are performed to characterize the crystal evolution of LMNO before and after lithiation and delithiation (Figure 3c–n). For pristine LMNO, the selected area electron diffraction (SAED) pattern (Figure 3d) exhibits the typical diffraction spots of double perovskite structure. The HR‐TEM image in Figure 3e represents the typical ($02\bar{2}$) crystal plane of double perovskite material, and the interplanar crystal spacing is 0.276 nm, in accordance with the density functional theory (DFT) calculated 0.275 nm (**Table** **1**). TEM‐EDS elemental mapping images reveal that La, Mn, Ni, and O elements are homogeneously distributed in the LMNO particles. Correspondingly, SEM‐EDS data verified the same conclusion (Figure S3, Supporting Information). In order to prepare lithiation‐LMNO, Li|LMNO cells are discharged to 0.01 V (Li^+^ insertion process). As shown in Figure 3h,i, SAED pattern (viewed along zone axis [$11\bar{1}$]) of lithiation‐LMNO suggests the typical diffraction spots of double perovskite structure, and the interplanar crystal spacing of (022) is 0.285 nm, which represent interplanar crystal spacing increasing slightly after Li‐ion insertion. This expansion of crystal plane is confirmed by DFT calculations (Table 1). From EDS mapping images of lithiation‐LMNO (Figure 3j), it is obvious that the particles maintain structural integrity, and LMNO particle was not reduced and decompose into various oxides (e.g., La_2_O_3_, Mn_2_O_3_, MnO_2_, and NiO). Perovskite (ABO_3_) type SmNiO_3_ was reported that a Li^+^ insertion cause the reduction of B‐Site Ni valence (Ni^3+^ to Ni ^2+^).^[^ ^45^ ^]^ Meanwhile, Quanli Hu et al. proposed that there are reversible redox reactions from the conversion between Mn^3+^ and Mn^4+^ in RMnO_3_ material families (R = La, Nd, Sm, Eu).^[^ ^46^ ^]^ Similarly, when a Li^+^ insert into LMNO, B‐site Mn or Ni ion may be reduced to low valence. Combined with XPS analysis data (Figure S1d–f, Supporting Information), the possible reaction process was proposed that Mn^4+^ was reduced to Mn^3+^ after Li^+^ insert into LMNO. After Li‐ion desertion (charge to 3 V), as shown in Figure 3l,m, SAED pattern (viewed along zone axis [$03\bar{1}$]) of delithiation‐LMNO displays the typical diffraction spots of perovskite structure, and the interplanar crystal spacing of (002) is 0.382 nm (DFT calculated value is 0.389 nm, Table 1), which represent interplanar crystal spacing reducing slightly (resuming to pristine state) after Li‐ion desertion and the double perovskite structure without obvious change. EDS mapping images (Figure 3n) exhibit the structural integrity of delithiation‐LMNO after ion insertion/extraction. Also, delithiation‐LMNO sample displays the similar XPS spectra as pristine LMNO sample after ion insertion and desertion (Figure S1a–c,g–i, Supporting Information). Considering these points, it could be believed that Li ion insertion/extraction process of LMNO is reversible and the double perovskite frame structure remains stable.

**Table 1:** The lattice parameters (*a*, *b*, and *c*), the interplanar crystal spacing (*d*_022_ and *d*_002_), the unit‐cell volume (*V*) and intercalation energy (Δ*E*_Li_) of LMNO with and without Li‐ion intercalation

System	a [nm]	b [nm]	c [nm]	*d*_022_ [nm]^a)^	*d*_002_ [nm]	V [nm^3^]	Δ*E*_Li_ [eV]
La_8_Mn_4_Ni_4_O_24_	0.777	0.777	0.777	0.275	0.387	0.469	0
La_8_Mn_4_Ni_4_O_24_ + Li	0.790	0.790	0.788	0.280	0.395	0.491	−1.47
La_8_Mn_4_Ni_4_O_24_ + 16Li	0.842	0.842	0.842	0.298	0.421	0.574	−0.63

^a)^ Note that in double‐perovskite crystalline, the spacing of (022) crystal plane is equal to that of (202), ($02\bar{2}$) and ($\bar{2}20$).

**Figure 4:**
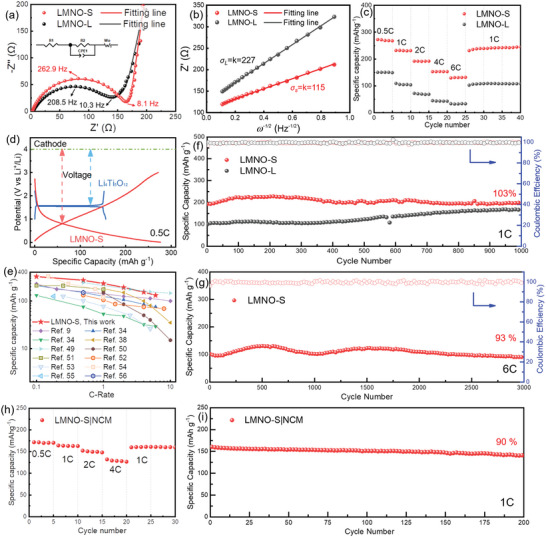
Electrochemical performances of Li|LMNO‐S and Li|LMNO‐L half cells and LNMO‐S|LCO full cells. a) Nyquist plots of LMNO‐S and LMNO‐L, the inset is equivalent circuit. b) Plots of Z′ versus *ω* ^−1/2^ after 1 cycle. c) Rate capability of LMNO‐S and LMNO‐L. d) Comparison of charging–discharging profiles between LMNO‐S and commercial LTO at 0.5C. e) Comparing plot of specific capacity versus C‐rate between our work and published data. f) Long cycling performances of LMNO‐S and LMNO‐L at 1C. g) Long cycling performances of LMNO‐S at 6C. h) Rate capability of LMNO‐S|NCM full cell. i) Long cycling performances of LMNO‐S|NCM full cell at 1C.

Additionally, the electrochemical performance of Li|LMNO half cells has been further researched. Cyclic voltammetry test (CV) was carried out with a potential range of 0.01–3.0 V at a scan rate of 0.2 mV s^−1^. In the first half scanning cycle (from 3.0 to 0.01 V), the curve shows a large and broad reduction peak, ranging from 0.9 to 0.01 V, and the peak disappeared in the subsequent cycles (Figure S4, Supporting Information). This phenomenon can be ascribed to formation of SEI layer and electrolyte reduction in the first cycle, which is correspond to the charging–discharging curves (Figure 3a). Electrochemical impedance spectroscopy (EIS) was employed to evaluate Li^+^ transport and diffusion behaviors. The LMNO electrodes consist of LMNO powders, carbon black and carboxymethylcellulose (CMC) with a weight ratio of 8:1:1 (see Experimental Section for details). **Figure** **4**a shows the Nyquist plots of Li|LMNO‐S and Li|LMNO‐L half cells, where intersections with the abscissa axis at the high frequency area present to the electrolyte resistance (*R*_0_), the semicircles at middle frequencies correspond to the overlapping of the SEI film (*R*_SEI_) and charge transfer resistance (*R*_ct_), and the straight line at low frequencies is attributed to the Warburg diffusion (*W*_o_) of Li^+^ in the electrodes. In order to compare diffusion process of two materials, Nyquist plots of LMNO‐S and LMNO‐L (Figure S5, Supporting Information) were obtained after 1 cycle (discharge to 0.01 V from OCV and charge to 3 V). The Li‐ion diffusion coefficient (D_Li_^+^) could be calculated based on the diffusion process at low frequency area in Nyquist plots (Figure 4b, and Figure S5, Supporting Information) by the following equation:^[^ ^38^, ^47^, ^48^ ^]^

(1) $$Z_{\mathrm{re}}=R_{\mathrm{ct}}\;+R_{b}+\sigma \omega^{-\frac{1}{2}}$$

(2) $$D=\frac{R^{2}T^{2}}{2n^{4}F^{4}A^{2}c^{2}\sigma^{2}}\;$$

where *R* is the gas law constant, *T* is the thermodynamic temperature, *n* is the electron number per molecule according to the electronic transfer reaction, *F* is the Faraday constant, *A* is the surface area of the LMNO electrode, and *c* is the max Li‐ion concentration in LMNO electrode. *σ* is the Warburg factor, which was calculated from the slope of fitting lines in Figure 4b, real part of the impedance spectra (*Z*_re_) versus reciprocal square root of angular frequency (*ω^−1/2^*) and Equation (1). The slope (Warburg factor, *σ*) of LMNO‐S and LMNO‐L are *σ*_s_ = 115 and *σ*_L_ = 227, respectively. Putting *σ* into the Equation (2), the Li‐ion diffusion coefficients were calculated as *D*_s_ = 2.54 × 10^−14^ and *D*_L_ = 4.71 × 10^−15^ cm^2^ s^−1^. Therefore, the diffusion velocity of Li‐ion is much faster in LMNO‐S electrode than that in LMNO‐L, which was verified by the superior rate capability of LMNO‐S in Figure 4c. In addition, specific surface area (SSA) of two samples was measured by N_2_ adsorption method. As shown in Figure S6, Supporting Information, the specific surface area (SSA) value of LMNO‐S (2.7 m^2^ g^−1^) is larger than that of LMNO‐L (0.9 m^2^ g^−1^). Also, the electronic conductivity of LMNO was measured as 0.50 S cm^−1^ by calculating from EIS and direct current polarization methods (Figure S7, Supporting Information). High electronic conductivity indicates fast electron transport in electrode and shows potential advantage in rate capability of the LMNO anode.

The fast‐charging performances were evaluated by C‐rate test ranged from 0.5C to 6C after activation processes at 0.1C (Figure 4c). During the cycles with C‐rate of 0.5C, 1C, 2C and 4C, high specific capacities of 271, 232, 191 and 153 mAh g^−1^ are observed. When the current density increases to 6C (1200 mA g^–1^), corresponding to a 10 min fast charging–discharging process, there are still retaining the specific capacity of 130 mAh g^–1^. Galvanostatic charge and discharge (GCD) curves (versus Li metal) of LMNO‐S, LMNO‐L and commercial Li_4_Ti_5_O_12_ (LTO) are compared in Figure 4d. When comparing with the LTO anode, LMNO‐S shows lower operating voltage of 0.8 V and a higher specific capacity of >270 mAh g^–1^. If assembling full cells with the same cathode, the output voltage and energy density of LMNO full cell will markedly exceed those of LTO full cells. Figure 4e exhibits the plot of specific capacity versus C‐rate for comparing our work with other published perovskite type anodes.^[^ ^9^, ^34^, ^38^, ^49^, ^50^, ^51^, ^52^, ^53^, ^54^, ^55^, ^56^ ^]^ Comparatively, LMNO‐S displays higher rate capability than most reported values. As given in Figure 4f, an activation process was observed during the cycle process at 1C, showing a slowly growing and then reducing tendency of specific capacity (from initial value of 192 mAh g^−1^ to maximum value of ≈230 mAh g^−1^). After 1000 cycles, a high specific capacity of 198 mAh g^−1^ was achieved, with the cycle retention of 103% (versus initial value).

**Figure 5:**
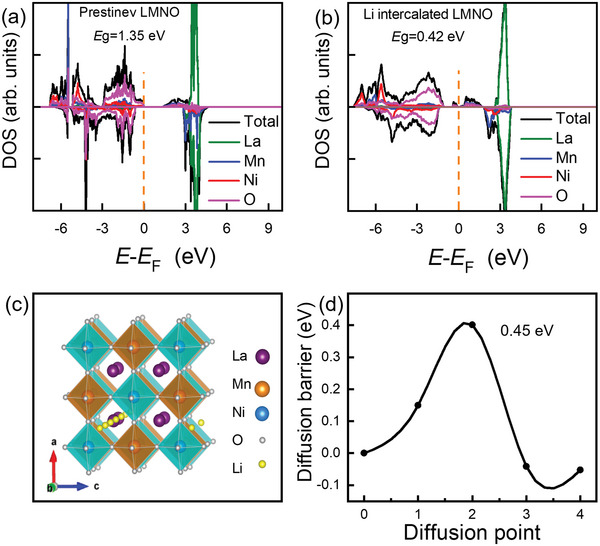
Electronic structures and Li migration barrier of LMNO. a) Projected density of states (PDOS) of pristine LMNO. b) PDOS of one Li intercalated LMNO. c) LMNO crystal structure and the Li^+^ diffusion path with the lowest barrier along [102]. d) Li diffusion energy with the lowest barrier along [102].

Moreover, long cycle test at 6C was further performed to verify the fast‐charging performance of LMNO‐S (Figure 4g). An initial specific capability of 99 mAh g^−1^ was obtained. After 3000 cycles, LMNO‐S shows an ultrahigh cycle retention of 93%. Considering the good electronic conductivity of LMNO‐S (**Figure** **5**a,b, and Figure S7, Supporting Information), a high percent of active material in electrode (active material:carbon black:CMC = 96:2:2) was prepared by the same method, and C‐rate test and long‐term cycle test were employed to evaluated their electrochemical performance (Figures S8 and S9, Supporting Information). Li|LMNO‐S half cells (active material = 96%, named as LMNO‐S 96 wt%) show the specific capacity of 158, 136 and 64 mAh g^−1^ at 0.5C, 1C and 6C. After 3000 cycles at 6C, Li|LMNO‐S 96 wt% cells exhibit a specific of 70 mAh g^−1^ with a high retention of 100%. Furthermore, full cell performances of LMNO‐S were studied by matching with LiNi_0.8_Co_0.1_Mn_0.1_O_2_ (NCM) cathode. The pre‐lithiation process was carried out in Li|LMNO‐S cells before full cell test. C‐rate and long cycle tests were employed to evaluate the electrochemical performance of LMNO‐S|NCM full cells. As shown in Figure 4h, the cells display the specific capacities of 171.3, 163.3, 151.9 and 131.8 mAh g^−1^ at 0.5C, 1C, 2C and 4C (1C = 180 mA g^−1^). When back to 1C, a high specific capacity of 160.7 was obtained, revealing the excellent rate capability. In addition, the LMNO‐S|NCM full cells represent high specific capacity and good cycling stability (200 cycles, retention = 90%) at 1C after the activation processes.

Furthermore, DFT calculations were performed to attain deep insight to understand the Li^+^ insertion/extraction mechanism. Considering the electronic conductivity is proportional to the inverse of bandgap, projected density of states (PDOS) was calculated to determine the bandgap of pristine LMNO.^[^ ^33^ ^]^ According to the PDOS data (Figure 5a), the bandgap of pristine LMNO was calculated as 1.35 eV, which is much lower than the bandgap of the reported electrode materials, such as 3.8 eV of spinel Li_4_Ti_5_O_12_,^[^ ^57^ ^]^ 2.1 eV of perovskite‐type Li_0.5_La_0.5_TiO_3_^[^ ^9^ ^]^ and 2.06 eV of perovskite‐type LiYTiO_4_,^[^ ^33^ ^]^ suggesting the higher electronic conductivity of LMNO and agreeing well with experimental result (Figure S7, Supporting Information). After incorporation of one Li^+^, LMNO displays a narrower band gap of 0.42 eV (Figure 5b) as compared with the pristine LMNO. Therefore, the Li intercalated LMNO demonstrates better electronic conductivity than the pristine system. It can be interpreted that the Li intercalation injects more electrons into the LMNO perovskite frameworks and then increases the carrier concentrations, which is confirmed by the shifting‐up of Fermi level in Figure 5b. The nudged elastic band calculations were carried out to study possible Li^+^ diffusion mobility in LMNO along different directions. Figure 5c,d shows that the Li^+^ diffusion energy barrier is 0.45 eV along the crystallographic direction of [102], which is lower than 0.68 eV along [101], 0.66 eV along [112] and 0.69 eV along [101_] (Figure S10, Supporting Information). Meanwhile, the PDOS and intercalation energy (Δ*E*_Li_) of fully lithiated‐LMNO were investigated. It was found that the bandgap disappears upon fully lithium‐ion insertion (Figure 5a,b, and Figure S11, Supporting Information). This phenomenon indicates that LMNO turns into a metal from a semiconductor after the Li^+^ insertion, which adequately facilitates the electron transport and charge transfer in lithiation‐LMNO electrode. The semiconductor‐metal transfer phenomenon was also discussed in several published papers (lithiated TiNb_2_O_7_,^[^ ^58^ ^]^ lithiated LiYTiO_4_^[^ ^33^ ^]^). Intercalation energy was calculated as −0.63 eV, further verifying the structural stability of lithiation‐LMNO (Table 1). These calculations indicate that excellent conductivity and intrinsic low Li ion diffusion barrier of double perovskite LMNO enables the fast Li^+^ transportation and high‐rate performance.

## 3 Conclusion

In summary, double perovskite LMNO was successfully synthesized by traditional solid‐state reaction method. In situ XRD and HR‐TEM characterization reveal that the reversible Li^+^ insertion/extraction process occurs in double perovskite type LMNO without any structural destruction. With a safety potential of 0.8 V versus Li^+^/Li, LMNO material as anode exhibits long cycle life and good rate capability. In C‐rate test, LMNO shows high specific capacity of 271 and 130 mAh g^−1^ at 0.5C and 6C. Especially, the Li|LMNO cells perform excellent long cycling retention of 103% after 1000 cycles at 1C and 93% after 3000 cycles at 6C, respectively. Additionally, theoretical calculations further demonstrate the high electronic conductivity, low diffusion energy barrier and structural stability of the pristine LMNO and lithiation‐LMNO. This work opens the door for exploring double perovskite oxides with promising lithium storage properties and the as‐prepared LMNO exhibits great potential as low‐voltage and high‐rate anode material for high‐performance LIBs.

## 4 Experimental Section

### Synthesis of La_2_MnNiO_6_ Powder

La_2_MnNiO_6_ was prepared via solid state method. 0.02 mol La_2_O_3_ (Aladdin), 0.01 mol Mn_2_O_3_ (Aladdin), and 0.02 mol NiO (Aladdin) were added into alcohol and ball milled (FRITSCH, pulverisette 7) for 6 h with a speed of 600 rpm, and the mass ratio of ball:powders:alcohol was 2:1:1. After ball‐milling and drying in 60 °C oven, the mixture was calcinated at various temperatures (1200 and 1400 °C) for 3 h at a heating rate of 5 °C min^−1^. After natural cooling and grinding, La_2_MnNiO_6_ (LMNO) fine powders were got.

### Fabrication of La_2_MnNiO_6_ Electrodes

La_2_MnNiO_6_ powder, carbon black (ECP‐600JD, Lion Specialty Chemicals Co. Ltd.) and carboxymethylcellulose (CMC, MTI Co. Ltd) were mixed well with a weight ratio of 8:1:1 and then dispersed in deionized water to form a slurry by using a mixer (Thinky, ARE‐310). The slurry was cast‐coated onto copper foil with an adjustable doctor knife. Then the foil was transferred into a drying oven at 60 °C for several hours and vacuum oven overnight. The drying copper foil was cut into small disks with a diameter of 12 mm.

### Fabrication of LiNi_0.8_Co_0.1_Mn_0.1_O_2_ Electrodes

Similarly, LiNi_0.8_Co_0.1_Mn_0.1_O_2_ (NCM, MTI Co. Ltd) powder, Super P (ECP‐600JD, Lion Specialty Chemicals Co. Ltd.) and Polyvinylidene Fluoride (PVDF, MTI Co. Ltd) with a weight ratio of 8:1:1 were mixed and dispersed in *N*‐methyl‐2‐pyrrolidinone (NMP) to form slurry using the mixer (Thinky, ARE‐310). Then slurry was cast‐coated onto aluminum foil and drying in vacuum oven at 100 °C overnight. The drying foil was punched into small disks with a diameter of 12 mm as a cathode in LMNO|NCM full cells.

### Materials Characterization

Crystalline phase structure was determined by X‐ray diffractometry (XRD, Bruker D8 Advance, Cu K*α* radiation) ranged from 10° to 80° (0.02° 2*θ* step, 6° min^−1^). In situ XRD data were performed by using X‐ray diffractometry coupled with electrochemical workstation (Bio‐Logic, VMP‐300). The microstructure, morphology, and chemical compositions of the samples were characterized by SEM (JEOL JSM‐7800F) and TEM (JEM‐2100 Plus, JEM‐F200) equipped with EDS. Surface chemical composition data were obtained from XPS (ThermoFisher Scientific, ESCALAB 250Xi).

### Electrochemical Measurements

Electrochemical performance of LMNO electrodes was measured in Li|LMNO half cells. The CR2032 coin cells were prepared in an argon‐filled glovebox (O_2_ and H_2_O content less than 1 ppm), in which lithium metal (Adamas‐beta) was used as reference electrode and counter electrode, polymer membrane (Celgard 2325) was used as separator, and 1 mol L^−1^ LiPF_6_ dissolved in ethylene carbonate/diethyl carbonate (EC/DEC, v/v = 1/1) was used as liquid electrolyte. For LMNO|NCM full cell test, the liquid electrolyte was 1.0 mol L^−1^ LiPF_6_ dissolved in carbonate/dimethyl carbonate (EC/DMC, v/v = 1/1) with 2% vinylene carbonate (VC). Electrochemical behavior in cells was evaluated by using EIS technique and cyclic voltammetry (CV) with an electrochemical workstation (Bio‐Logic, VMP‐300). For EIS test, the perturbation bias voltage was 10 mV, operating frequency ranged from 7 MHz to 0.1 Hz. CV was carried out with a potential range of 0.01–3.0 V at a scan rate of 0.2 mV s^−1^. The galvanostatic charge and discharge (GCD) long cycle test and rate performance test (0.01–3.0 V for Li|LMNO cells, 2.0–4.2 V for LMNO|NCM full cells) were performed at room temperature on a battery testing system (LAND, CT2001A). Lithiation‐LMNO was obtained after discharge to 0.01 V at the first cycle in Li|LMNO cells and then washed by DEC solvent. Delithiation‐LMNO was prepared by the same process as lithiation‐LMNO except that cells were discharged to 0.01 V and then charged to 3 V.

### Storage Mechanism

The specific capacity *C* (mAh g^−1^) and storage mechanism were obtained based on experimental data and the following equation:^[^ ^32^, ^59^ ^]^

(3) $$C\;=\frac{nF}{3.6\;\times \;M}\;$$

where *n* is the exchanged Li^+^ number per unit cell. F is Faraday constant, 96 485 C mol^−1^. *M* is the molecular weight of La_2_MnNiO_6_, 487.4 g mol^−1^. With 1 Li^+^ intercalation per unit La_2_MnNiO_6_ cell, the theoretical specific capacity was ≈55 mAh g^−1^. For LMNO‐S electrode, experimental specific capacity of ≈300 mAh g^−1^ was observed at 0.1C (1C = 200 mA g^−1^ in this manuscript), hence the practical number of Li^+^ intercalation per unit La_2_MnNiO_6_ cell was calculated as 5.5 (Li/La mole ratio was 2.75). Therefore, the possible lithium storage mechanism was given as follow (based on LaMn_0.5_Ni_0.5_O_3_):

(4) $$2.75{\mathrm{Li}}^{+}+2.75e^{-}+{\mathrm{LaMn}}_{0.5}{\mathrm{Ni}}_{0.5}O_{3}\to {\mathrm{Li}}_{2.75}{\mathrm{LaMn}}_{0.5}{\mathrm{Ni}}_{0.5}O_{3}$$

Furthermore, Li/La mole ratio was confirmed by ICP‐OES measurement of lithiation‐LMNO sample. Before the measurement, lithiation‐LMNO electrodes were washed by diethyl carbonate (EDC) solvent to remove the lithium salt. As a result, Li/La mole ratio of 3.67 was obtained, higher than 2.75 (determined by electrochemical measurement at 0.1C). The higher mole ratio might be due to residual Li in the SEI layer and irreversible Li ion insert in perovskite.

### DFT Calculations

Calculations were calculated through density functional theory (DFT) in the Vienna ab initio simulation package (VASP).^[^ ^60^, ^61^ ^]^ The potentials were of the projector augmented wave (PAW) type, and the exchange–correlation part was treated within the generalized gradient approximation (GGA) of Perdew–Burke–Ernzerhof (PBE).^[^ ^62^, ^63^ ^]^ Considering of strong electron‐electron correlation effect,^[^ ^64^ ^]^ the Hubbard parameter *U* was introduced as *U* − *J* = 3.9 and 6.6 eV for Mn and Ni 3d states, respectively. The structural and electronic properties were calculated in the primitive cell of LMNO consisting of 40 atoms (8 La, 4 Mn, 4 Ni, and 24 O). The plane‐wave cutoff energy of 400 eV and a 3 × 3 × 3 k‐point grid was employed for geometry optimization, and a 6 × 6 × 6 k‐point grid for density of states (DOS). The energetically favorable sites of Li interstitial were determined by screening 52 possible sites in the LMNO. The intercalation energy (Δ*E*_Li_) was computed by the formula Δ*E*_Li_ = *E*_Li+LMNO_ − *E*_LMNO_ − *E*_Li_, where *E*_Li+LMNO_ and *E*_LMNO_ are the total energies of LMNO with and without Li‐ion intercalation. *E*_Li_ is the energy of the isolation Li atom. The intercalation energy was used to check the thermodynamic stability of Li in LMNO. The energy barriers of Li ion migration were calculated by the nudged elastic band method.

## Conflict of Interest

The authors declare no conflict of interest.

## Author Contributions

C.Z. conceived the experiment and carried out data analysis. W.L. supervised all aspects of the research. Y.Z. acquired the HRTEM images and SAED patterns. Z.N. and C.W. acquired the XPS spectrum. Y.Z. and Z.N. assisted in data analysis. Y.C. and Y.G. perform the DFT calculation and analysis. C.Z., Y.C., and W.L. wrote this paper. All the authors discussed the results and commented on the manuscript.

## Supporting information

Supporting Information

## Data Availability

The data that support the findings of this study are available from the corresponding author upon reasonable request.
