# Supplementary material for: Double Perovskite La2MnNiO6 as a High‐Performance Anode for Lithium‐Ion Batteries
Source: Adv Sci (Weinh). 2023 Apr 21;10(18):2300506. doi: 10.1002/advs.202300506 (PMC10288226; doi:10.1002/advs.202300506)
Supplement: Supplementary file 1 — Supporting Information [file ADVS-10-2300506-s001.pdf]

ADVANCED SCIENCE
Open Access

Supporting Information

for *Adv. Sci.*, DOI 10.1002/advs.202300506

Double Perovskite La_2MnNiO_6 as a High-Performance Anode for Lithium-Ion Batteries

Chang Zhang, Yue Zhang, Zhiwei Nie, Cong Wu, Tianyi Gao, Nan Yang, Yi Yu, Yuanyuan Cui, Yanfeng Gao and Wei Liu**

Supporting Information

Double perovskite La_2MnNiO_6 as a high-performance anode for lithium-ion batteries

Chang Zhang[1], Yue Zhang[1], Zhiwei Nie[1], Cong Wu[1], Tianyi Gao[1], Nan Yang[1], Yi Yu[1,2], Yuanyuan Cui[3], Yanfeng Gao[3], and Wei Liu[1,2,4]**

1. C. Zhang, Y. Zhang, Z. Nie, C. Wu, T. Gao, Prof. N. Yang, Prof. Y. Yu, and Prof. W. Liu
School of Physical Science and Technology, ShanghaiTech University, Shanghai, 201210, P. R. China
E-mail: liuwei1@shanghaitech.edu.cn

2. Prof. Y. Yu, and Prof. W. Liu
Shanghai Key Laboratory of High-resolution Electron Microscopy, ShanghaiTech University, Shanghai, 201210, P. R. China

3. Prof. Y. Cui, and Prof. Y. Gao
School of Materials Science and Engineering, Shanghai University, Shanghai, 200444, P. R. China
E-mail: cui-yy@shu.edu.cn

4. Prof. W. Liu
Key Laboratory of Advanced Energy Materials Chemistry (Ministry of Education)
Nankai University, Tianjin, 300071, P. R. China

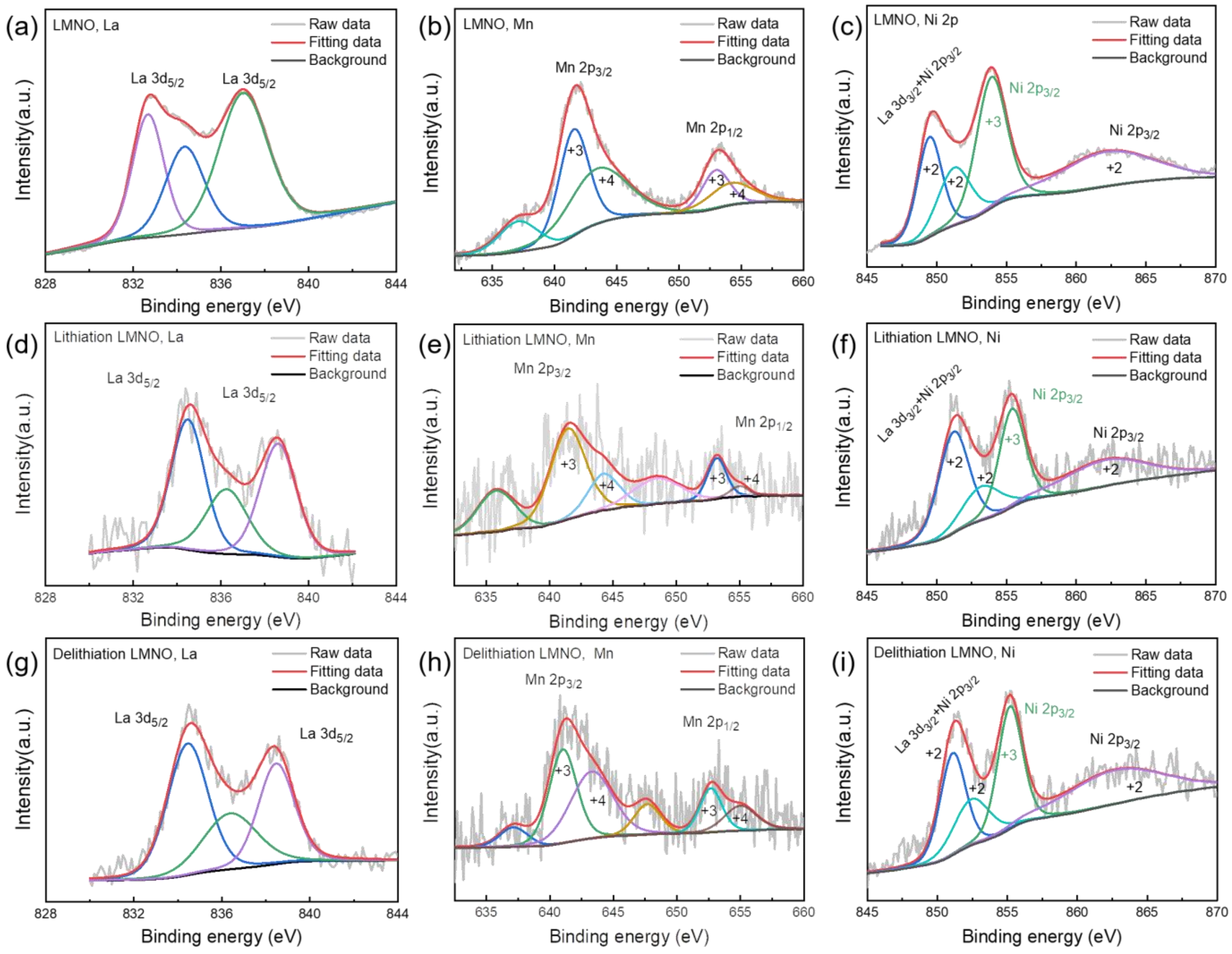

Figure S1. XPS analysis of samples. XPS spectrum of La 3d, Mn 2p and Ni 2p region of LMNO (a-c), lithiation-LMNO (d-f) and delithiation-LMNO (g-i), respectively.

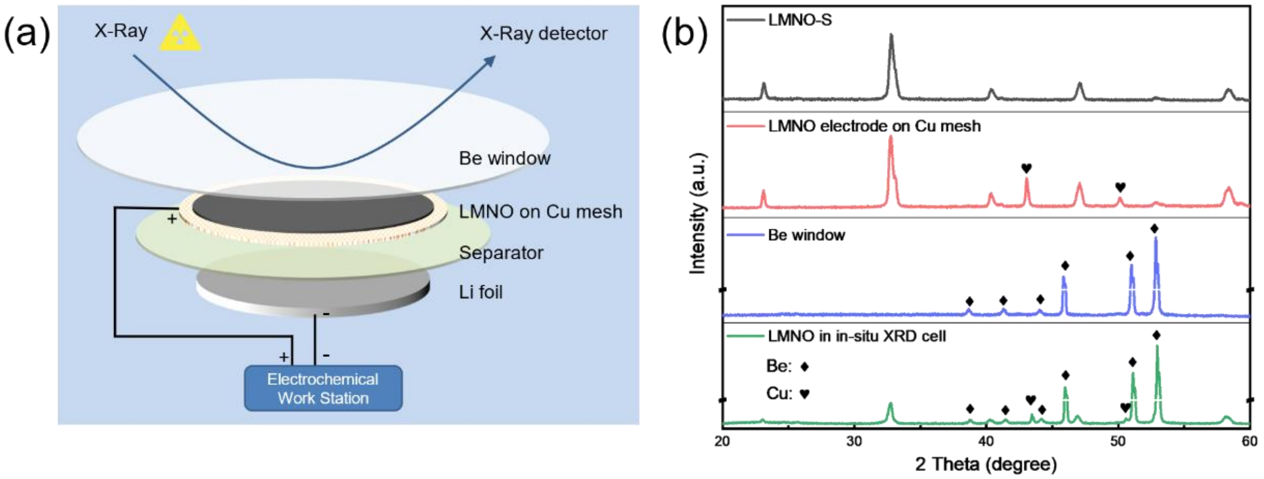

Figure S2.(a) Schematic illustration of in-situ XRD cell. (b) XRD patterns of LMNO-S powder, LMNO electrode, Be window and LMNO electrode in in-situ XRD cell.

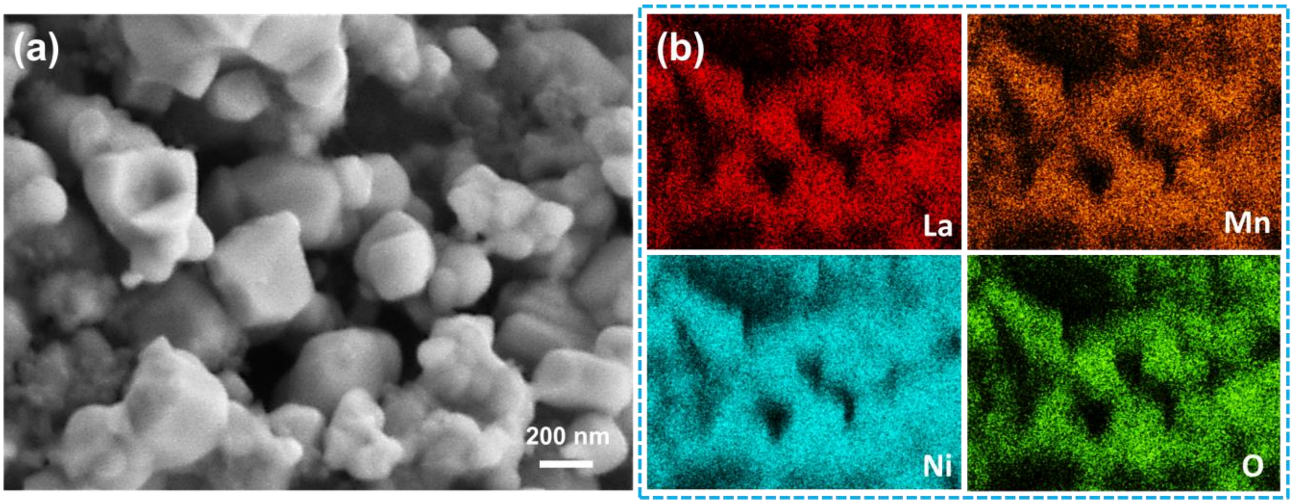

Figure S3.SEM image of LMNO-S and corresponding EDS elemental mapping images of La, Mn, Ni and O elements.

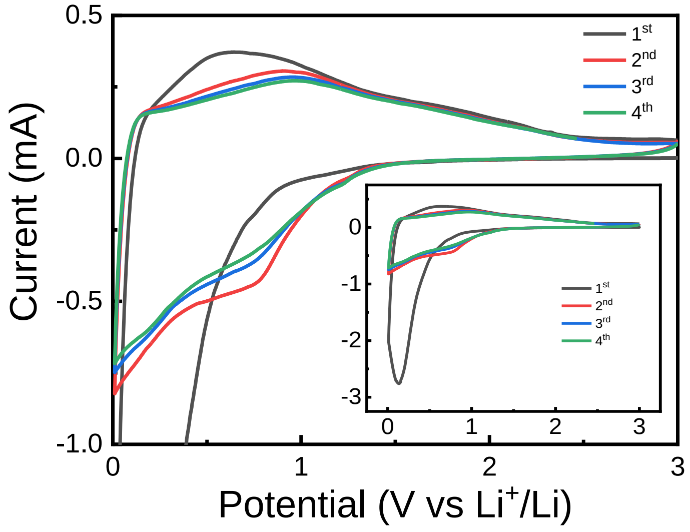

Figure S4. Cyclic voltammetry curves of Li|LMNO cell with a potential range of 0.01-3.0 V.

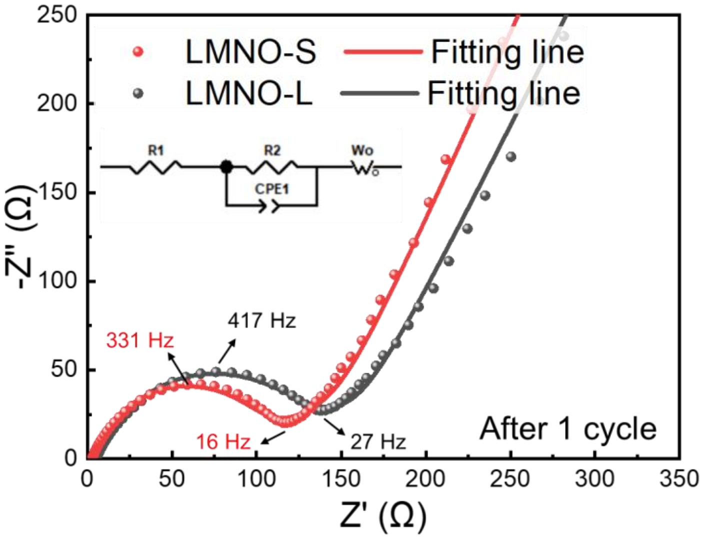

Figure S5. Nyquist plots of LMNO-S and LMNO-L after 1 cycle, the inset is equivalent circuit.

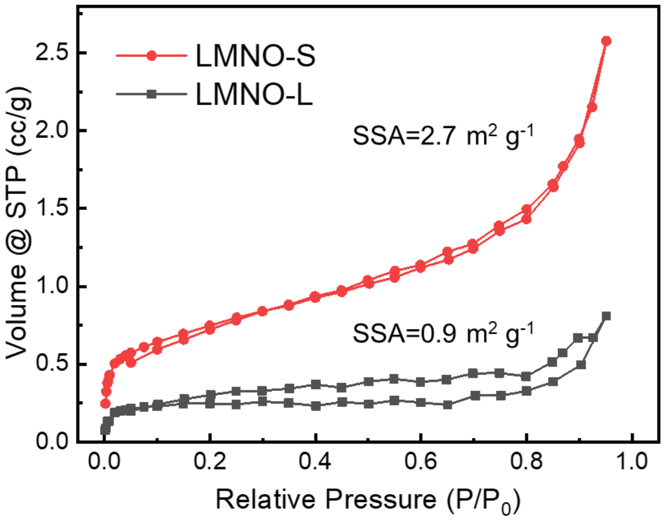

Figure S6. N_2 adsorption and desorption isotherms of LMNO-S and LMNO-L.

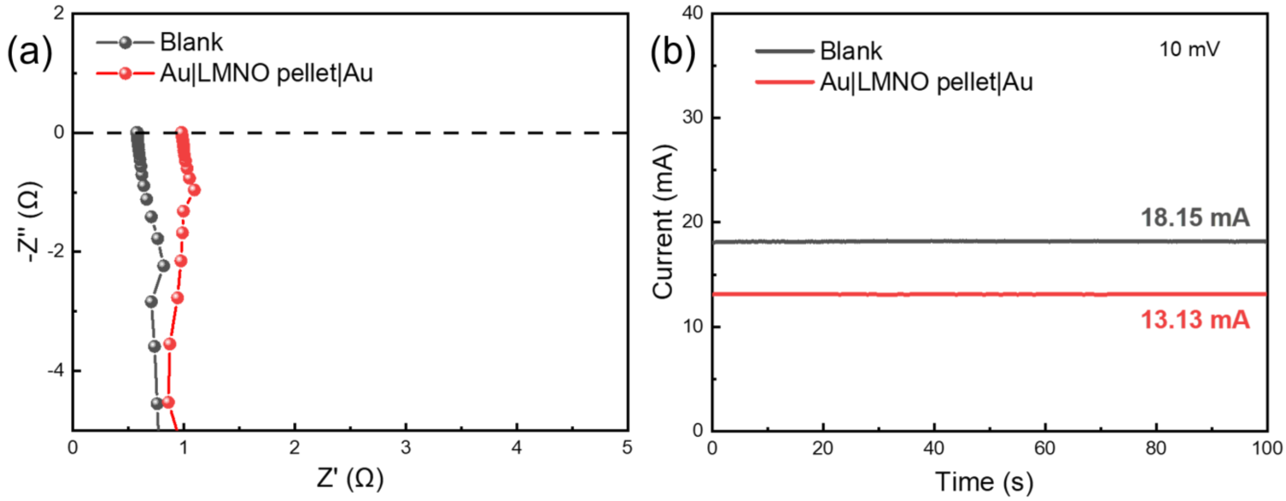

Figure S7. (a), Nyquist plots of LMNO-S pellet. (b), Direct current polarization plots of LMNO-S pellet at 10 mV.

The LMNO fine powder was transferred into a stainless-steel die and pressed, and then the green pellets were sintered at high temperature. The diameter and thickness of LMNO pellet are d=1.1 cm and L=0.1 cm. EIS result represents that the LMNO-S pellet sample is the good electronic conductor. Direct current polarization data reveal that the ohm resistance of LMNO-S is 0.21 Ω. Hence, the

electronic conductivity σ_e is calculated as 0.50 S cm^{-1} according to equation S2, much higher than ionic conductivity of liquid electrolyte in cell (10^{-2}~10^{-3} S cm^{-1}).

$$R = \rho \frac{L}{S} \tag{S1}$$

$$\sigma_e = \frac{1}{\rho} = \frac{L}{RS} = \frac{4L}{R\pi d^2} \tag{S2}$$

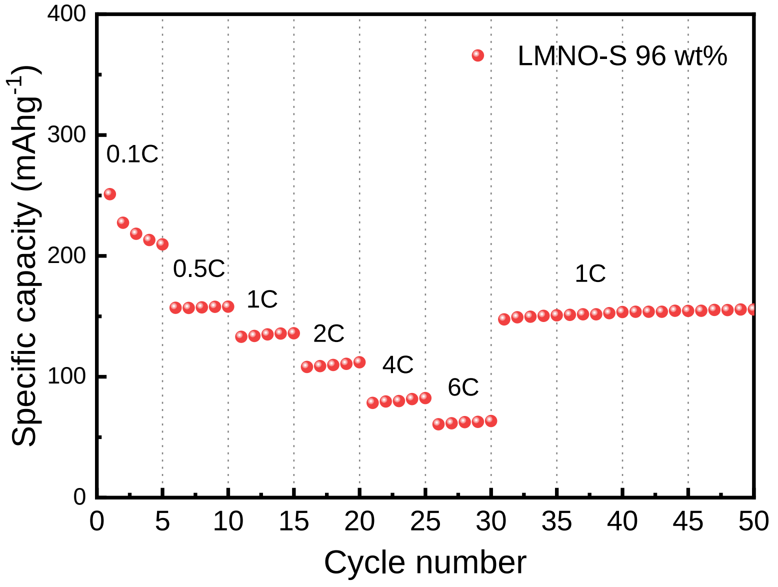

Figure S8. Rate capability of LMNO-S|Li half-cell, in which percent of active material is 96%.

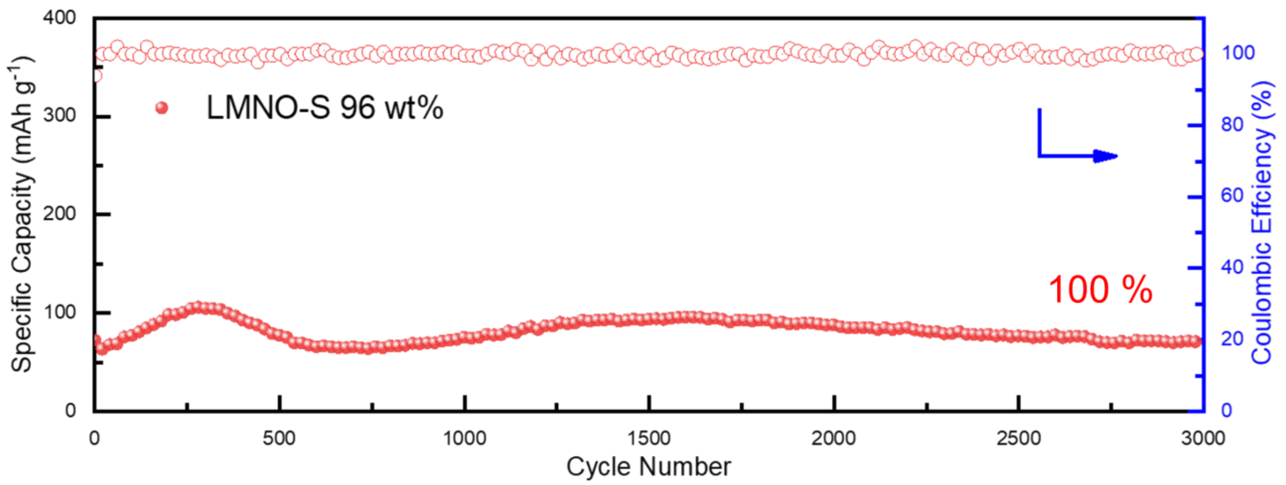

Figure S9. Long cycle performance of LMNO-S|Li half-cell at 6C, in which percent of active material is 96%.

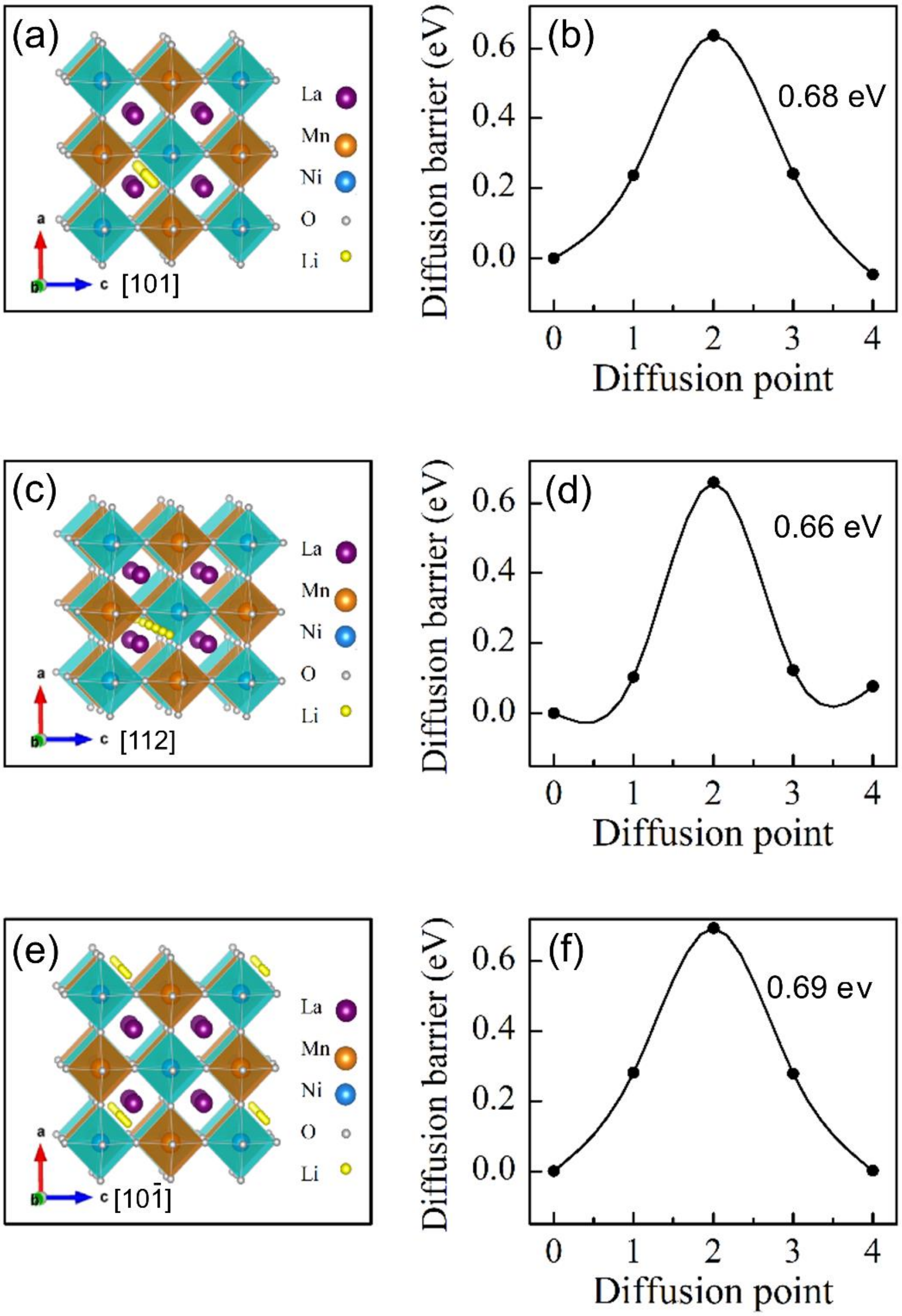

Figure S10. (a) LMNO crystal structure and the Li^+ diffusion path along [101]. (b) Li diffusion energy along [101]. (c) LMNO crystal structure and the Li^+ diffusion path along [112]. (d) Li diffusion energy along [112]. (e) LMNO crystal structure and the Li^+ diffusion path along [10$\bar{1}$]. (f) Li diffusion energy along [10$\bar{1}$].

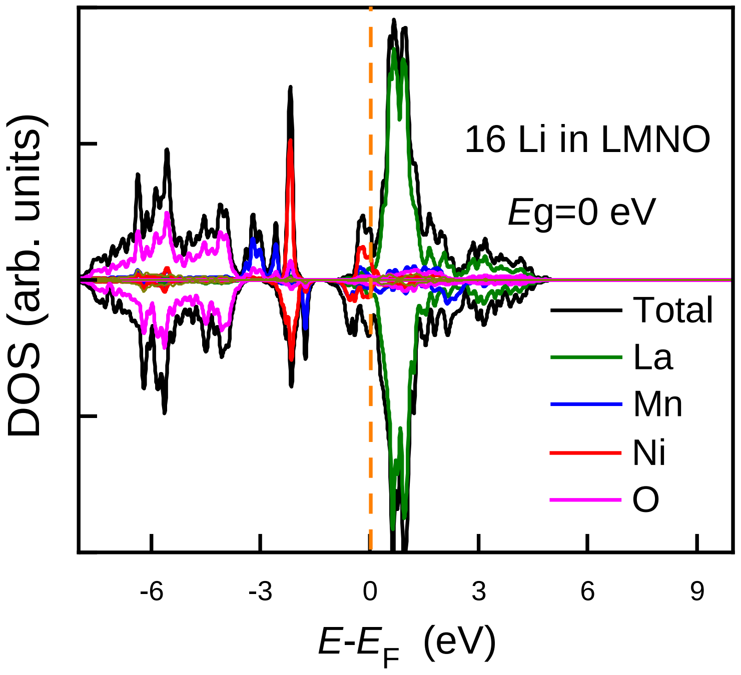

Figure S11. PDOS of Li fully intercalated LMNO.
